# Molecular and subregion mechanisms of episodic memory phenotypes in temporal lobe epilepsy

**DOI:** 10.1093/braincomms/fcac285

**Published:** 2022-11-05

**Authors:** Robyn M Busch, Lamis Yehia, Ingmar Blümcke, Bo Hu, Richard Prayson, Bruce P Hermann, Imad M Najm, Charis Eng

**Affiliations:** Epilepsy Center, Neurological Institute, Cleveland Clinic, 9500 Euclid Avenue, Cleveland, OH 44195, USA; Department of Neurology, Neurological Institute, Cleveland Clinic, 9500 Euclid Avenue, Cleveland, OH 44195, USA; Genomic Medicine Institute, Lerner Research Institute, Cleveland Clinic, 9500 Euclid Avenue, Cleveland, OH 44195, USA; Genomic Medicine Institute, Lerner Research Institute, Cleveland Clinic, 9500 Euclid Avenue, Cleveland, OH 44195, USA; Epilepsy Center, Neurological Institute, Cleveland Clinic, 9500 Euclid Avenue, Cleveland, OH 44195, USA; Institute of Neuropathology, University Hospitals Erlangen, Schwabachanlage 6, D–91054 Erlangen, Germany; Quantitative Health Sciences, Lerner Research Institute, Cleveland Clinic, 9500 Euclid Avenue, Cleveland, OH 44195, USA; Department of Neurology, Neurological Institute, Cleveland Clinic, 9500 Euclid Avenue, Cleveland, OH 44195, USA; Department of Anatomic Pathology, Cleveland Clinic, 9500 Euclid Avenue, Cleveland, OH 44195, USA; Department of Neurology, University of Wisconsin, 1685 Highland Avenue, Madison, WI 53705, USA; Epilepsy Center, Neurological Institute, Cleveland Clinic, 9500 Euclid Avenue, Cleveland, OH 44195, USA; Department of Neurology, Neurological Institute, Cleveland Clinic, 9500 Euclid Avenue, Cleveland, OH 44195, USA; Genomic Medicine Institute, Lerner Research Institute, Cleveland Clinic, 9500 Euclid Avenue, Cleveland, OH 44195, USA; Taussig Cancer Institute, Cleveland Clinic, 9500 Euclid Avenue, Cleveland, OH 44195, USA; Department of Genetics and Genome Sciences, Case Western Reserve University School of Medicine, 10900 Euclid Avenue, Cleveland, OH 44106, USA

**Keywords:** temporal lobe epilepsy, memory, spatial transcriptome, targeted spatial protein profiling, RNA-Seq

## Abstract

Memory dysfunction is prevalent in temporal lobe epilepsy, but little is known about the underlying pathophysiological etiologies. Here, we use spatial quantitation to examine differential expression of targeted proteins and transcripts in four brain regions essential for episodic memory (dentate gyrus, CA3, CA1, neocortex) between temporal lobe epilepsy patients with and without episodic memory impairment. Brain tissues were obtained from dominant temporal lobectomies in 16 adults with pharmacoresistant temporal lobe epilepsy associated with hippocampal sclerosis. Verbal memory tests from routine pre-operative clinical care were used to classify episodic memory as impaired or intact. Digital spatial profiling of a targeted protein panel and the whole transcriptome was performed using tissue sections from the temporal neocortex and hippocampus. We performed differential expression and pathway enrichment analysis between the memory groups within each temporal lobe region. Several proteins associated with neurodegenerative disease were overexpressed in the neocortex of patients with impaired memory, corroborating our prior findings using bulk transcriptomics. Spatial transcriptomics identified numerous differentially expressed transcripts in both neocortical and hippocampal subregions between memory groups, with little overlap across subregions. The strongest molecular signal was observed in the CA3 hippocampal subregion, known to play an essential role in memory encoding. Enrichment analyses revealed BDNF as a central hub in CA3-related networks regulating phenotype-relevant processes such as cognition, memory, long-term potentiation and neuritogenesis (*P_adj_* < 0.05). Results suggest memory impairment in temporal lobe epilepsy with hippocampal sclerosis is associated with molecular alterations within temporal lobe subregions that are independent from hippocampal cell loss, demographic variables and disease characteristics. Importantly, each temporal subregion shows a unique molecular signature associated with memory impairment. While many differentially expressed transcripts and proteins in the neocortex have been associated with neurodegenerative disorders/processes, differentially expressed transcripts in hippocampal subregions involve genes associated with neuritogenesis and long-term potentiation, processes essential for new memory formation.

## Teaser

Busch *et al.* employed digital spatial profiling to demonstrate that memory impairment in temporal lobe epilepsy is related to unique molecular alterations within different subregions of the hippocampus and temporal neocortex that are independent from seizure frequency/severity and the presence of hippocampal sclerosis.

## Graphical Abstract

**Graphical Abstract:**
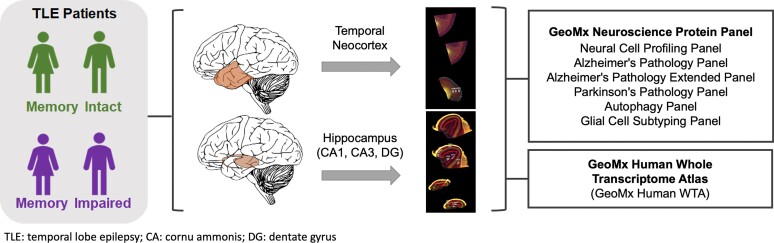

## Introduction

Temporal lobe epilepsy (TLE) is the most common type of focal epliepsy and is associated with high risk for memory deficits, which patients report as one of the most concerning aspects of their condition.^1,2^ Memory deficits are most prevalent in those with pharmacoresistant TLE and hippocampal sclerosis (HS).^3^ However, a large subset of patients with TLE and HS paradoxically have intact memory performance, even with histopathological evidence for marked hippocampal cell loss. Unfortunately, despite the high prevalence of memory impairment in TLE, very little is known about the underlying pathophysiological etiologies.^4,5^

Temporal lobe structures play a crucial role in episodic memory, with regional specificity of the hippocampal subfields and surrounding cortical areas.^6,7^ Animal and human studies, including studies in TLE, have demonstrated that the dentate gyrus (DG) and cornu ammonis sector 3 (CA3) are primarily involved in encoding and early retrieval of episodic memories. In contrast, cornu ammonis sector 1 (CA1) plays a primary role in delayed retrieval, consolidation and recognition of episodic memories. These three hippocampal regions form a trisynaptic circuit in which granule cells in the DG project to pyramidal cells in CA3 that project to pyramidal cells in CA1 and then out to basal and lateral neocortical temporal regions via the subiculum. Hence, memory is governed by a highly orchestrated, morphologically controlled process. Importantly, loss of granule cells in the DG and pyramidal cells in hippocampal subfields are hallmarks of HS and are associated with episodic memory impairment.^8^

Our recent findings using classical bulk RNA sequencing showed genes associated with neurological functions are underexpressed in the temporal neocortex of TLE patients with impaired memory compared to those with intact memory and implicated genes involved in the pathogenesis of neurodegenerative disorders (e.g. *APOE*, *APP, MAPT*) in memory impairment in TLE.^9^ However, what remains unclear is whether these molecular differences are specific to temporal lobe subregions essential for episodic memory function. We hypothesized that molecular changes associated with memory impairment in TLE are region-specific.

**Figure 1:**
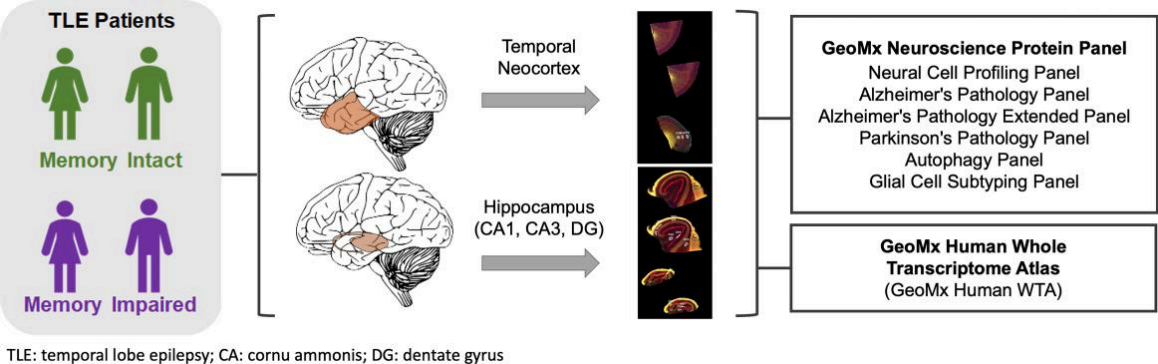
**Study design**.

The objectives of the current study were two-fold: (i) to measure levels of proteins associated with neurodegenerative disorders as a marker of memory impairment and (ii) to perform spatial molecular profiling of the temporal lobe from a well-matched and carefully clinically phenotyped group of patients with language-dominant TLE and HS in order to examine regional expression differences and uncover biomarkers for episodic memory impairment in TLE associated with HS (Fig. 1).

## Methods

### Participants

Brain tissue samples were obtained from 16 adults with pharmacoresistant TLE and HS who underwent left (language dominant) temporal lobe resections for treatment of their seizures. The surgery typically includes resection of the temporal lobe anterior to the vein of Labbe (temporal pole, middle and inferior temporal gyri, and the basal temporal region) with removal of the mesial structures (hippocampus and amygdala). Language dominance was determined with functional MRI or Wada testing (*n* = 8). In patients without a language lateralization procedure, right-handedness in conjunction with seizure semiology (i.e. postictal aphasia) and/or preoperative cognitive test results (i.e. verbal memory and/or language deficits) was used as a proxy for left-sided language dominance (*n* = 8). Given the known association between *APOE* polymorphisms and memory performance (i.e. *APOE* ε4 detrimental, *APOE* ε2 protective), only patients who were homozygous for *APOE* ε3 allele (wildtype) were included in the study. Anti-seizure medication regimens remained stable between the time of neuropsychological testing and surgery. H&E stained brain tissue slides were reviewed by a clinical neuropathologist with expertise in epilepsy (I.B.), and a diagnosis of HS ILAE Type 1 confirmed for all samples.^8^ Tissue and clinical data used in this study were obtained from an IRB-approved epilepsy tissue bank and clinical data registry at the Cleveland Clinic. Patients with a history of prior resective neurosurgery were excluded.

### Memory measures and classification

All patients completed a comprehensive neuropsychological evaluation approximately 3.5 months (SD = 3 months) prior to surgery that included assessment of verbal episodic memory. Specifically, patients completed measures of both story recall (Logical Memory subtest of the Wechsler Memory Scale—Third or Fourth Edition) and word-list learning (Rey Auditory Verbal Learning Test or Wechsler Memory Scale—Third Edition). Tests were administered according to their respective test manuals and scored using demographically corrected norms. All scores were transformed into standard scores (mean = 100; SD = 15), and patients were separated into two memory groups based on a mean composite delayed memory score (combined delayed story recall and word-list learning tasks).^10,11^ Patients with scores less than 85 were classified as having ‘impaired’ memory (*n* = 8) and those with mean standard scores of 85 or above were classified as having ‘intact’ memory (*n* = 8). As intended, the impaired memory group demonstrated significantly lower delayed verbal memory scores (median 4th percentile) than the intact memory group (median 37th percentile).

### Tissue preparation and region of interest selection

Formalin-fixed, paraffin-embedded tissue sections were placed onto glass slides and stained with a cocktail of standard morphology markers consisting of either oligo-tagged antibodies (protein) or oligo-tagged in situ hybridization probes (RNA) to perform digital spatial profiling. To enable the histological identification of the tissues, all slides were stained with HuR (neurons), IBA1 (microglia), MBP (myelin), as well as SYTO™ 13 (Thermo Fisher Scientific, Waltham, MA, USA) nuclear staining (DNA). These stains were selected to help maximize our ability to clearly identify neuronal cells in the tissue samples. The stained slides were scanned into the GeoMx Digital Spatial Profiling platform (NanoString, Seattle, WA, USA). Initial ROIs were selected by an epileptologist (I.M.N.) and then reviewed by a neuropathologist (I.B.) to verify anatomical selection of each ROI prior to analysis. For each patient, three primary hippocampal regions were identified (i.e. DG, CA3, CA1) along with three primary neocortical regions in the temporal lobe (i.e. cortical layers II and III, layer IV, layers V and VI). The specimens were not collected prospectively; however, based on our clinical practice for preparing pathology specimens, the neocortical specimens in this study are likely to have come from the fusiform or inferior temporal gyri. Specific regions of interest (ROIs) were selected within each of the identified anatomical regions for downstream molecular profiling. ROIs were drawn manually around HuR stained neurons within each anatomical region. Only regions with at least 40 000 µm2 and at least 100 cells were selected to ensure proper probe quantification.

### Molecular profiling of prioritized ROIs

Targeted protein analysis was conducted using a comprehensive neuroscience protein assay including Neural Cell Profiling, Alzheimer’s Pathology (plus extended module), Parkinson’s Pathology, Autophagy, and Glial Cell Subtyping panels (NanoString, Seattle, WA, USA). Whole transcriptome analysis was performed using the GeoMx Human Whole Transcriptome Atlas (NanoString). Differential expression between memory groups was examined using the GeoMx Digital Spatial Profiler Analysis Suite version 2.5.0.144 (NanoString), which integrates modules for quality control, data normalization, and statistical comparisons.^12^ For the comprehensive neuroscience protein assay, data were normalized to the geometrical mean of negative control immunoglobulins (mouse IgG2a, mouse IgG1, and rabbit IgG). For the Whole Transcriptome Atlas, ROI quality control (QC) detected raw read counts of at least 133 337 reads, aligned read counts of at least 114 781 reads and a minimum sequencing saturation of 97.53%. Probe QC detected zero outliers. The limit of quantification (LOQ) was defined as the GeoMean(NegProbes) × GeoSD(NegProbes),^2^ where GeoMean is the geometric mean, NegProbes are the negative probes, and GeoSD is the geometric standard deviation. We applied target filtering to retain targets with read counts above the LOQ in each ROI. This resulted in 10 707 genes (out of 18 000+). We then applied Q3 normalization on the filtered ROIs and gene targets. For comparisons where each patient had multiple ROIs within each anatomical region, we applied a linear mixed model adjusting for patient ID as the random effects variable. For comparisons including each anatomical subregion (DG, CA3, CA1, neocortex), where each patient has only one sample/ROI, we used the Student’s *t*-test with a significant statistical level of *P* < 0.05.

### Statistical analyses and analytical strategy

Baseline descriptive statistics stratified by memory group (intact versus impaired) were calculated. Statistics are presented as means with standard deviations for normally distributed variables and medians with interquartile ranges for non-normally distributed variables. Independent samples t-tests, Wilcoxon rank-sum, or Fisher’s exact tests were used to examine group differences on demographic and disease-related variables. *P* values of <0.05 were considered statistically significant.

Digital spatial profiling comparisons were performed on temporal neocortex and hippocampal tissue from patients with TLE, a subset of whom were in our prior study,^8^ using the comprehensive neuroscience protein assay described above. In order to identify new biomarkers for memory impairment in TLE, we performed spatial whole transcriptome analyses using collective ROIs for the two major temporal lobe regions (i.e. temporal neocortex, hippocampus) and then separately for each anatomical subregion (i.e. DG, CA3, CA1, cortical layers II and III, layer IV, layers V and VI) between memory groups. For comparisons including the temporal neocortex and hippocampus, where each patient has multiple subregions per anatomical region, we applied a linear mixed model adjusting for patient ID as the random effects variable. For comparisons including each anatomical subregion (DG, CA3, CA1, cortical layers), where each patient has only one sample/ROI, we applied the *t*-test. In all analyses, *P* values of <0.05 were considered statistically significant.

### Pathway enrichment analysis

Ingenuity Pathway Analysis (IPA, Qiagen Bioinformatics, Redwood City, CA, USA) was used to perform pathway/network analysis and enrichment for diseases and functions for the transcriptome analyses. Inputs consisted of differentially expressed genes (*P* < 0.05) with and without log2 fold changes greater than +1 and less than −1. All outputs are reported with *P_adj_* < 0.05 considered statistically significant.

### Role of the funding source

The funding sources for this study had no role in design of this study, collection, analysis, or interpretation of study data, preparation of the manuscript, or the decision to submit the paper for publication.

### Ethical publication statement

We confirm that we have read the Journal’s position on issues involved in ethical publication and affirm that this report is consistent with those guidelines.

### Data availability

The data that support the findings of this study are not publicly available because of IRB-based restricted access, but further information about the datasets is available from the corresponding author on reasonable request.

## Results

### Participants

On average, patients were 41 years old (range 24–54) and had 13 years of education (range 11–18) at baseline assessment. Age at seizure onset was 26 years on average (range 7–46), with mean duration of epilepsy of 15 years (range 5–31). All patients in this sample were White and non-Hispanic, and over half the sample was male (56%). The two memory groups were well-matched on demographic and most disease-variables. The only exception was longer epilepsy duration (19 versus 11 years) in the intact memory compared to the impaired memory group (Table 1).

**Table 1:** Demographic and epilepsy-related data for study patients

	Intact memory (*n* = 8)	Impaired memory (*n* = 8)	*P*
	Mean (SD) or *Median (IQR)	Mean (SD) or *Median (IQR)	
Age	40.63 (8.63)	41.75 (10.7)	0.820
Education*	12 (12–15)	12 (12–13)	0.954
Age at seizure onset	21.6 (10.9)	30.5 (11.9)	0.143
Duration of epilepsy (years)	19.0 (8.1)	11.3 (4.5)	0.034
Seizure frequency (per month)*	6.1 (1.3–36.3)	4.3 (3.6–12.8)	0.958
Number of anti-seizure medications	2.1 (0.8)	1.9 (0.6)	0.513
Full scale IQ	95.0 (5.4)	88.9 (11.6)	0.196
Mean verbal delayed memory	95.0 (8.9)	73.1 (6.0)	<0.001
	***Number*** **(%)**	***Number*** **(%)**	
Sex (male)	4 (50%)	5 (63%)	1.000
History of febrile seizures	5 (50%)	3 (38%)	0.619
History of GTCs	6 (75%)	4 (50%)	0.608
History of status epilepticus	1 (13%)	1 (13%)	1.000

SD = standard deviation; IQR = interquartile range; GTCs = generalized tonic-clonic seizures.

### Targeted protein analyses

**Figure 2:**
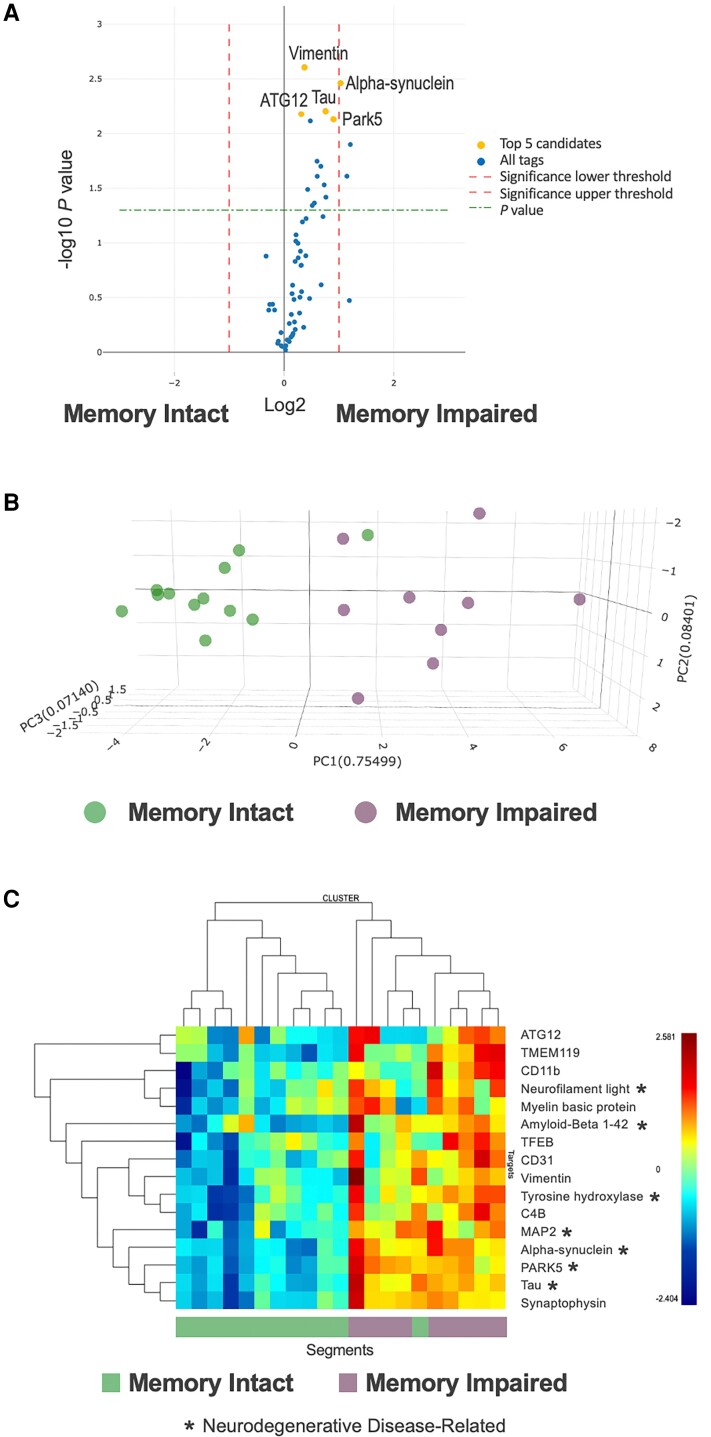
**Spatial memory-relevant protein expression in temporal neocortical tissues**. (**A**) Volcano plot of differential protein expression between TLE patients with and without memory impairment in the discovery series. (**B**) Principal components analyses (PCA) depicting differentially expressed proteins between TLE patients with and without memory impairment in the discovery series. (**C**) Heatmap depicting differentially expressed proteins between TLE patients with and without memory impairment in the discovery series.

Initial spatial protein expression experiments were conducted on neocortical temporal and hippocampal tissues from a subset of TLE patients who were also in our prior bulk RNA sequencing study (*n* = 8; 4 impaired memory, 4 intact memory).^8^ We identified 16 proteins that were differentially abundant (*P* < 0.05) in neocortical tissues of patients with impaired memory compared to those with intact memory (Fig. 2A). These markers clearly separated neocortical regions from TLE patients according to memory group (Fig. 2B), with significant differential expression in many proteins associated with neurodegenerative disorders (e.g. tau, alpha-synuclein, amyloid-beta 1-42, NEFL, MAP2, PARK5) (Supplementary Table 1), similar to our bulk RNA sequencing findings.^9^ More specifically, these proteins were overexpressed in neocortical regions from the memory impaired group compared to the memory intact group (Fig. 2C). A weaker signal was observed in hippocampal tissues, with only one differentially expressed protein (phospho-tau, *P* = 0.009) that was overexpressed in the memory impaired group (Supplementary Fig. 1A).

**Figure 3:**
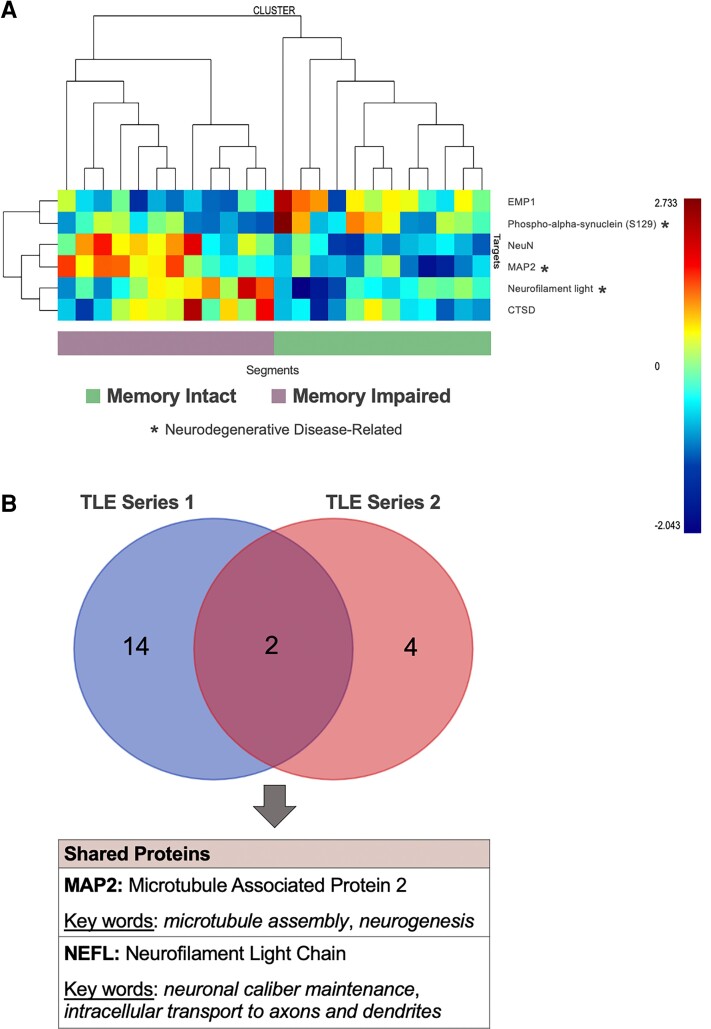
**Examination of memory-relevant proteins in the validation series.** (**A**) Heatmap depicting differentially expressed proteins between TLE patients with and without memory impairment in the validation series. (**B**) Overlap in differentially expressed proteins between the discovery and validation series. TLE = temporal lobe epilepsy.

Subsequent spatial protein experiments were conducted on temporal lobe tissue from a series of new, unrelated patients with TLE (n = 8; 4 impaired memory, 4 intact memory) to validate our findings. We identified six proteins (EMP1, phospho-alpha-synuclein S129, NeuN, MAP2, NEFL, CTSD), most of which were overexpressed (*P* < 0.05) in neocortical tissues of TLE patients with impaired memory compared to those with intact memory (Fig. 3A and Supplementary Table 2). MAP2 and NEFL were also identified in the discovery series of TLE patients (Fig. 3B). We identified three differentially expressed proteins (APP, amyloid-beta 1-42, BAG3) in the hippocampal tissues of patients in this validation series (*P* < 0.05), all of which were overexpressed in the memory impaired group (Supplementary Fig. 1B).

### Whole transcriptome analyses

**Figure 4:**
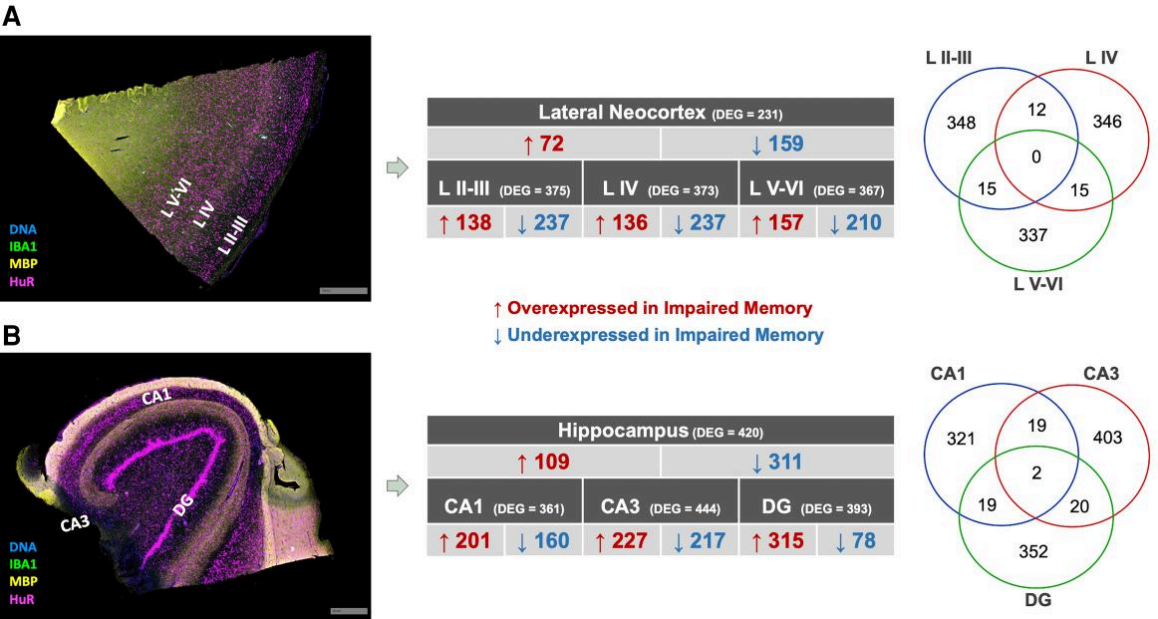
**Spatial whole transcriptome expression in temporal neocortical and hippocampal tissues.** (**A**) Representative image of immunofluorescent staining of temporal neocortex with three regions of interest: L II and III, L IV, and L V and VI, and summary of differential transcript expression between TLE patients with and without memory impairment in the neocortex and cortical subregions. (**B**) Representative image of immunofluorescent staining of the hippocampus with three regions of interest: DG, CA1, CA3, and summary of differential transcript expression between TLE patients with and without memory impairment in the hippocampus and hippocampal subregions. L II III = cortical layers II and III, L IV = cortical layer IV, L V and VI = cortical layers V and VI, DG = dentate gyrus, CA3 = cornu ammonis sector 3 and CA1 = cornu ammonis sector 1.

To examine our hypothesis that the molecular changes associated with memory impairment in TLE are region-specific and to identify possible biomarkers of memory impairment in an unbiased manner, our second set of experiments was designed to examine differentially expressed transcripts (DETs), using the whole transcriptome, in the same neocortical and hippocampal regions as well as in temporal lobe subregions essential for episodic memory. We interrogated tissues from all 16 unrelated TLE patients from our protein analyses (eight impaired memory and eight intact memory). As with the protein analyses, we first analysed the neocortical and hippocampal tissues as separate gross anatomical entities. Then, we conducted stratified analyses whereby gene expression was examined in each of the neocortical and hippocampal subregions independently. A summary of the number of DETs identified in each temporal lobe region is shown in Fig. 4A and B. Notably, the majority of DETs were underexpressed in the neocortex of patients with impaired memory compared to those with intact memory overall as well as within each of the cortical subregions (Supplementary Table 3). Interestingly, there was no overlap in DETs across the three cortical subregions (Fig. 4A). While the majority of DETs in the hippocampus were underexpressed in the memory impaired group when all regions were examined together, independent examination of the hippocampal subregions revealed that most DETs were overexpressed in the memory impaired group (Fig. 4B and Supplementary Table 4). Interestingly, despite over 1000 DETs across the hippocampal subfields of interest, there was overlap in only 2 DETs (*SORL1* and *ZFC3H1*) across the three subregions (Fig. 4B).

**Figure 5:**
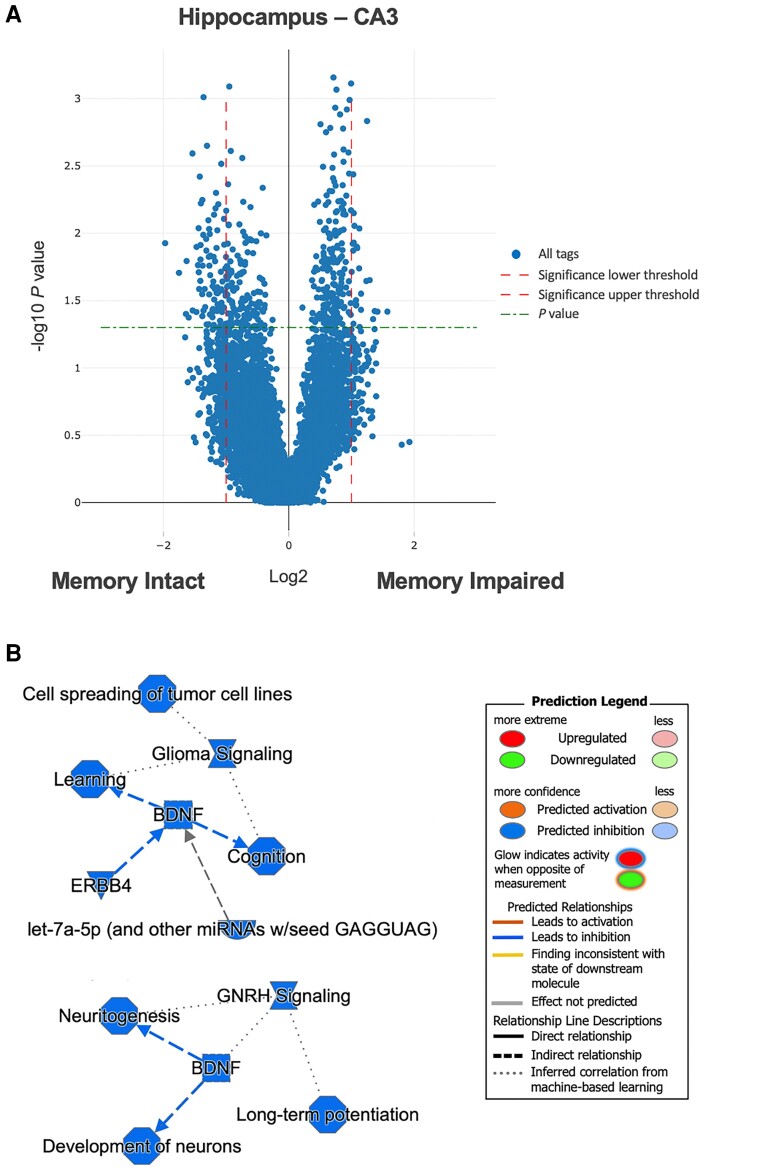
**Identification of differentially expressed genes from the hippocampus.** (**A**) Volcano plot of differential transcript expression in the CA3 region of the hippocampus between TLE patients with and without memory impairment. (**B**) Enrichment analyses of differentially expressed transcripts in the CA3 region of the hippocampus. Top network is derived from all DETs; bottom network is derived from DETs with log2 fold changes greater than 1 or less than −1. CA3 = cornu ammonis sector 3. Note that the legend may include predicted events not observed within the constructed networks.

**Figure 6:**
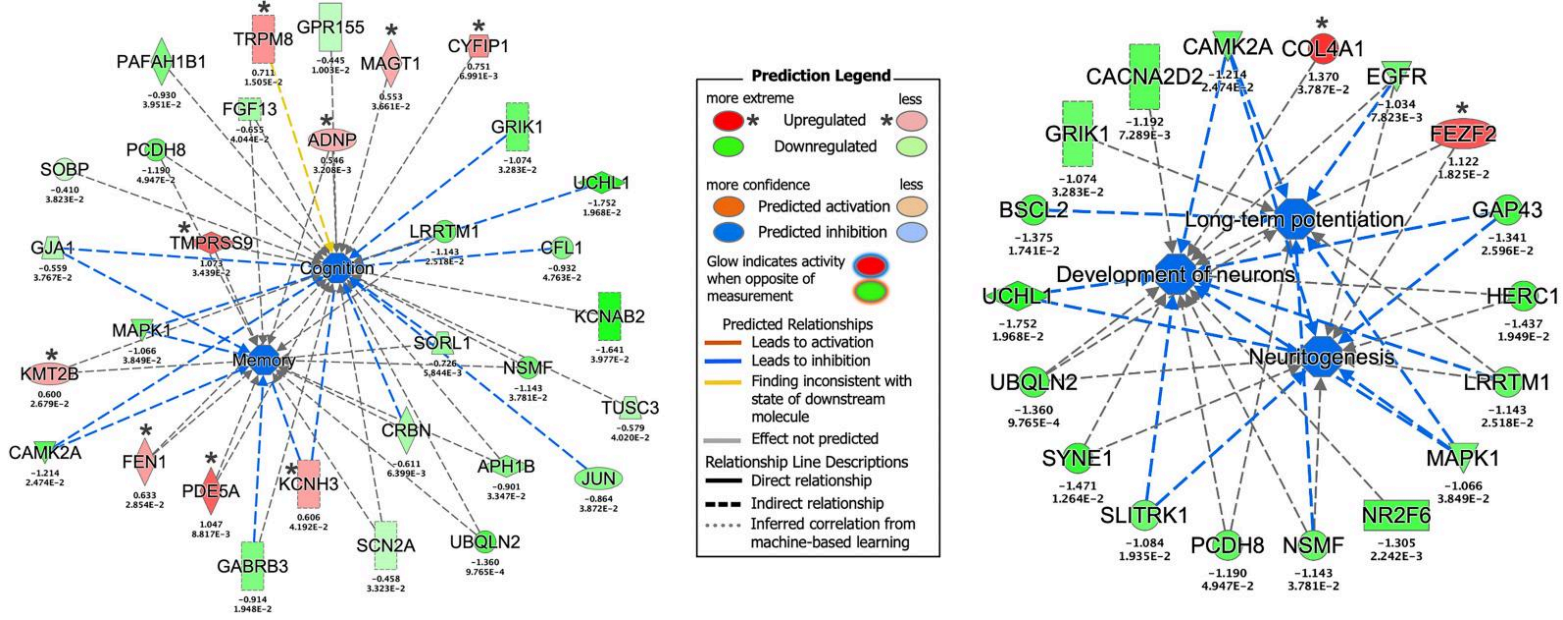
**Pathway enrichment analysis of differentially expressed genes from the hippocampus.** The left panel represents enrichment analysis of diseases and functions associated with the gene expression differences (all DETs) within the CA3 region. The right panel represents enrichment analysis of diseases and functions associated with the gene expression differences, derived from DETs with log2 fold changes greater than 1 or less than −1, within the CA3 region. CA3 = cornu ammonis sector 3. Note that the legend may include predicted events not observed within the constructed networks.

To identify biological pathways that could be impacted by the DETs, we performed pathway and functional enrichment analyses on neocortical temporal lobe and hippocampal regions and subregions (Supplementary Table 5). While there were hundreds of DETs within each temporal lobe subregion investigated, the strongest signal arose from the CA3 region of the hippocampus with 111 DETs with log2 fold changes greater than 1 or less than −1 (Fig. 5A). Relatedly, a machine learning algorithm implemented through Ingenuity Pathway Analysis (IPA) identified *BDNF* as a central hub in CA3-related networks regulating phenotype-relevant processes such as cognition, memory, long-term potentiation and neuritogenesis (*P_adj_* < 0.05; Fig. 5B). Analysis of the diseases and functions associated with the gene expression differences within the CA3 region from patients with and without memory impairments revealed ‘cognition’ (*P_adj_* = 2.6 × 10^−3^) and ‘memory’ (*P_adj_* = 2.1 × 10^−2^) as significant annotations (Fig. 6A***—***left panel), while analysis limited to genes with log2 fold changes greater than 1 or less than −1 revealed ‘long-term potentiation’ (*P_adj_* = 3.0 × 10^−3^), ‘development of neurons’ (*P_adj_* = 1.3 × 10^−2^) and ‘neuritogenesis’ (*P_adj_* = 4.1 × 10^−2^) (Fig. 6B***—***right panel).

## Discussion

This study is the first to employ spatial transcriptomics to elucidate the role of temporal lobe expression differences (protein and transcript) in memory impairment associated with TLE due to hippocampal sclerosis. Results demonstrate regional expression differences within the temporal lobe between TLE patients with and without episodic memory impairment. Across regions, we observe a common pattern of overrepresentation of differentially expressed proteins and transcripts involved in processes important for new memory formation (e.g. synaptic plasticity, long-term potentiation, neuritogenesis) and previously implicated in neurodegenerative disorders in the temporal lobe tissue of TLE patients with impaired memory. Intriguingly, these results challenge the existing notion that memory loss in mesial TLE is primarily due to neuronal cell loss associated hippocampal sclerosis.^13^ In this demographically and clinically well-matched series of adults with TLE and histopathologically confirmed HS, we demonstrate that the presence or absence of episodic memory impairment is associated with molecular alterations within subregions of the hippocampus and temporal neocortex that are unrelated to seizure frequency and severity (memory groups were well matched on disease-related variables) or the presence of HS (all patients in this study had hippocampal sclerosis, ILAE Type I). This observation is consistent with findings from the aging literature that suggest changes in learning and memory are due to alterations in synaptic physiology and aberrant cell signalling rather than neuronal loss within brain regions important for memory.^14^

Using classical bulk RNA sequencing, we have previously shown differential transcript expression in the temporal neocortex of patients with TLE with and without memory impairment, with an overrepresentation of genes in pathways associated with neurotransmitters and nervous system signalling, including many associated with neurodegenerative disorders, such as Alzheimer’s disease.^9^ Here, using a targeted protein panel, we demonstrate differential expression of several proteins associated with neurodegenerative diseases (e.g. tau, alpha-synuclein, amyloid-beta 1-42, NEFL, MAP2, PARK5) in the temporal neocortex of two independent series of patients with TLE, providing evidence that the transcriptional differences observed in our bulk RNA sequencing data are associated with downstream effects at the protein level. There was overlap in two proteins between our discovery and validation series (i.e. NEFL, MAP2). Importantly, these two proteins play a role in the mechanisms of long-term potentiation and synaptic plasticity,^15,16^ processes that are essential for learning and memory, including consolidation and long-term memory storage.^17,18^ NEFL has been extensively studied and found to be an important biomarker for neurodegenerative diseases that is inversely related to cognition (i.e. higher protein levels associated with poorer cognition).^16,19,20^ While MAP2 has been less widely studied in the context of neurodegenerative disorders, there is evidence to suggest it can serve as a marker of age-dependent deterioration of dendritic stability and plasticity^21^ and may play a role in the pathogenesis of tauopathies.^22^ Although it is not surprising to find neurologically relevant differentially expressed genes, finding those associated with memory-related processes is unlikely to be due to random chance. Additionally, we believe the weaker signal in the hippocampus compared to the lateral cortex may be explained by subregional differences in expression. This hypothesis could not be directly examined using the protein dataset due to the experimental design; however, we examined this with our transcriptome data, which indeed revealed strong subregional expression differences.

At the transcriptome level, we observed hundreds of differentially expressed transcripts in both neocortical and hippocampal tissues between individuals with and without memory impairment. Importantly, we demonstrate, for the first time, that differential transcript expression varies spatially across subregions essential for new memory formation (i.e. dentate gyrus, CA3, CA1, neocortex) as well as across different cortical layers in the neocortex of the anterior temporal lobe (i.e. cortical layers II and III, layer IV, layers V and VI) as a function of memory phenotype in TLE, with almost no transcript overlap across regions. This suggests that each subregion of the temporal lobe plays a unique role in the overall molecular signature associated with memory impairment. While there are several theories regarding the roles of specific neuroanatomical structures in episodic memory formation, it is clear that memories arise from a complex interaction between subregions of the hippocampus and temporal neocortical regions.^11,12^

While there were hundreds of DETs within every temporal subregion examined (range 231–444 DETs), the most robust findings were observed in the CA3 region of the hippocampus with 444 significant targets, 111 of which had log2 fold change values greater than +1 or less than −1. This is perhaps not surprising given memory impairment in this study was defined using measures that assess free recall of previously presented information (i.e. short stories, list words) after a relatively short delay (i.e. ∼20 minutes). It has been well established that the subregions of the hippocampus differ in terms of cellular composition, organization and connectivity^23,24^ and make unique contributions to the processes/stages of episodic memory.^7^ More specifically, the CA3 and DG are input structures that play a primary role in memory encoding, while the CA1 and subiculum are output structures that play a role in memory retrieval.^6,7,25^

Interestingly, a machine learning algorithm implemented through IPA identified *BDNF* as a central hub in CA3-related networks regulating, or responding to, phenotype-relevant processes such as cognition, memory, long-term potentiation and neuritogenesis. BDNF is a neurotrophin known to play an essential role in learning and memory by regulating synaptic transmission (excitatory and inhibitory) and activity-dependent plasticity and inducing long-term potentiation in the hippocampus.^26,27^ As a result, underexpression or overexpression of BDNF can negatively affect learning and memory,^26,27^ and numerous studies now seek to identify ways in which to alter BDNF expression (e.g. exercise, diet, supplements, transcranial magnetic stimulation) in an attempt to treat memory deficits associated with aging and neurodegenerative diseases.^26^

Enrichment analyses of DETs in the CA3 region of the hippocampus revealed an overrepresentation of transcripts involved in neuritogenesis, neuron development, long-term potentiation, learning and cognition, all consistent with our memory phenotype. In the past couple decades, a host of studies have confirmed the critical importance of the CA3 region for rapid encoding of new memories and linked synaptic plasticity within CA3 circuits to episodic memory.^28,29^ Unmyelinated axons, known as mossy fibres, project from granule cells in the DG to pyramidal cells in CA3, with remarkable capability for pre and postsynaptic plasticity to facilitate long-term potentiation required for memory encoding.^28^ Thus, it is reassuring that the CA3 region showed the strongest molecular signature in this study, albeit in the new context of TLE.

There are several study limitations that deserve mention. First, the sample size is relatively small, and findings will need to be replicated in larger patient cohorts, which will be challenging to attain in the short term. Nevertheless, we are encouraged that our observations at the protein level are consistent with transcript level findings and implicate similar pathways, diseases, and functions. In addition, the genes and pathways identified are biologically relevant to our memory phenotype and consistent with those identified in other neurological populations with impaired memory (e.g. Alzheimer’s disease). Of note, our careful study design (e.g. relatively homogenous cohort of individuals with dominant TLE and HS who are homozygous for the *APOE* ε3 allele and well matched on relevant demographic and disease variables) overcome the perceived small sample size, and the fact that differences observed were consistently identified would suggest a large effect size. Second, all patients in this study had pathologically confirmed hippocampal sclerosis (ILAE Type I). Thus, results may not generalize to nonlesional TLE or TLE associated with other neuropathologies. Nevertheless, our results suggest that memory impairment in TLE may be driven primarily by molecular alterations, independent of demographic or disease characteristics and neuropathological findings. The role of chronic seizures in the upregulation of the neurodegenerative genes and proteins and memory dysfunction in at risk TLE patients with no HS cannot be ruled out. Future research in more diverse patient cohorts (such as those with TLE and no HS who underwent temporal lobe resection based on direct intracranial recordings with SEEG) will be needed to confirm this observation.

In summary, our results indicate that variability in memory impairment associated with TLE and HS is associated with discrete molecular alterations in subregions of the hippocampus and temporal neocortex, most notably in genes associated with synaptic plasticity and long-term potentiation required to encode episodic memories as well as those associated with neurodegenerative processes that affect memory. Together, our findings suggest that there are a series of integrated and overlapping networks that contribute to memory impairment in TLE independent of demographic factors, disease-related variables, epilepsy severity markers, or hippocampal neuropathology and that each subregion of the temporal lobe appears to play a unique role in the overall molecular signature associated with memory impairment. These findings inform the growing interest in the overlap of epilepsy with neurodegenerative conditions.^30^ In addition, this work will serve as a proof-of-principle model for further investigations into the biological underpinnings of episodic memory dysfunction and other discrete cognitive abnormalities in non-neurodegenerative conditions more generally and may suggest potential biomarkers and/or biological targets for future therapy to improve memory performance.

## Supplementary Material

fcac285_Supplementary_Data

## Abbreviations

APOE =: apolipoprotein E
APP =: amyloid precursor protein
BAG3 =: BAG cochaperone 3
CA1 =: cornu ammonis 1
CA3 =: cornu ammonis 3
CTSD =: cathepsin D
DG =: dentate gyrus
EMP1 =: epithelial membrane protein 1
HuR =: human antigen R
HS =: hippocampal sclerosis
IBA1 =: allograft inflammatory factor 1 (AIF1)
MAP2 =: microtubule associated protein 2
MAPT =: microtubule associated protein tau
MBP =: myelin basic protein
NEFL =: neurofilament light chain
NEUN =: neuronal nuclear protein
PARK5 =: ubiquitin C-terminal hydrolase L1 (UCHL1)
TLE =: temporal lobe epilepsy
