# Supplementary material for: Molecular and subregion mechanisms of episodic memory phenotypes in temporal lobe epilepsy
Source: Brain Commun. 2022 Nov 5;4(6):fcac285. doi: 10.1093/braincomms/fcac285 (PMC9679425; doi:10.1093/braincomms/fcac285)
Supplement: fcac285_Supplementary_Data [file fcac285_supplementary_data.zip › Supplementary Figure 1 and Supplementary Tables 1 and 2.docx]

**Supplementary Material**

**Molecular and Subregion Mechanisms of Episodic Memory Phenotypes**

**in Temporal Lobe Epilepsy**

Robyn M. Busch, Ph.D., Lamis Yehia, Ph.D., Ingmar Blümcke, M.D., Bo Hu, PhD., Richard Prayson, M.D., Bruce P. Hermann, Ph.D., Imad M. Najm, M.D., Charis Eng, M.D., Ph.D.

**Table of Contents**

Supplementary [Figure 1: Differentially expressed proteins in hippocampal tissues from TLE patients 2](#_Toc96415955)

Supplementary [Table 1: Differentially expressed proteins in neocortical tissues from TLE patients (series 1) 3](#_Toc96415956)

Supplementary [Table 2: Differentially expressed proteins in neocortical tissues from TLE patients (series 2) 4](#_Toc96415957)

# **Supplementary Figure 1: Differentially expressed proteins in hippocampal tissues from TLE patients**

**A.**

**
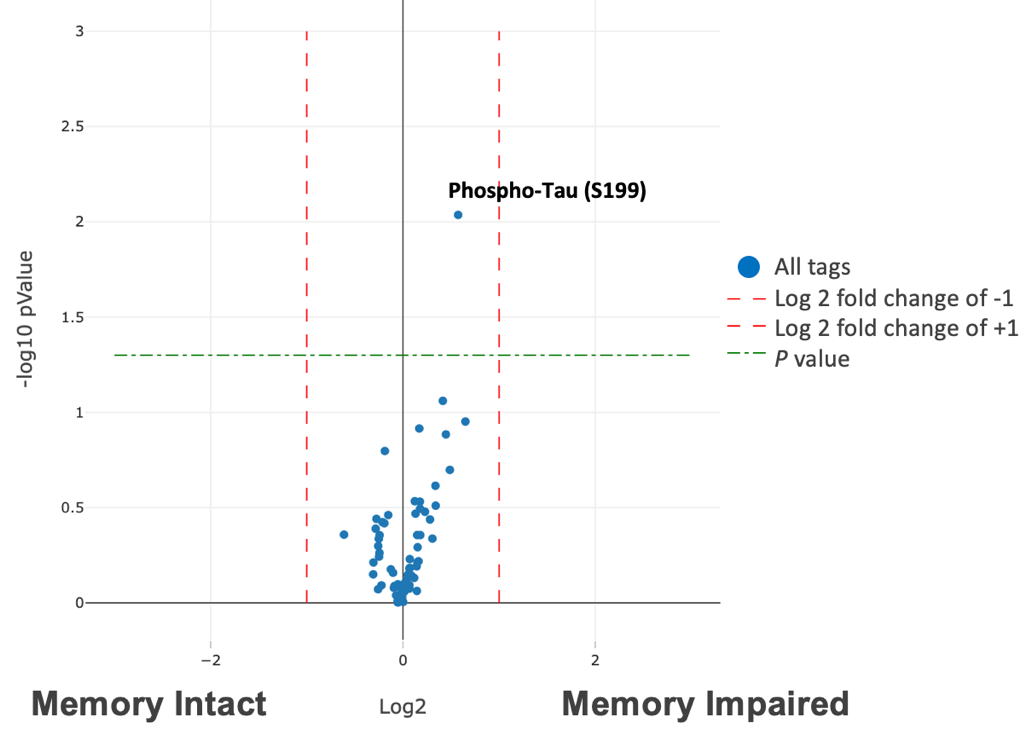
**

**B.**

**
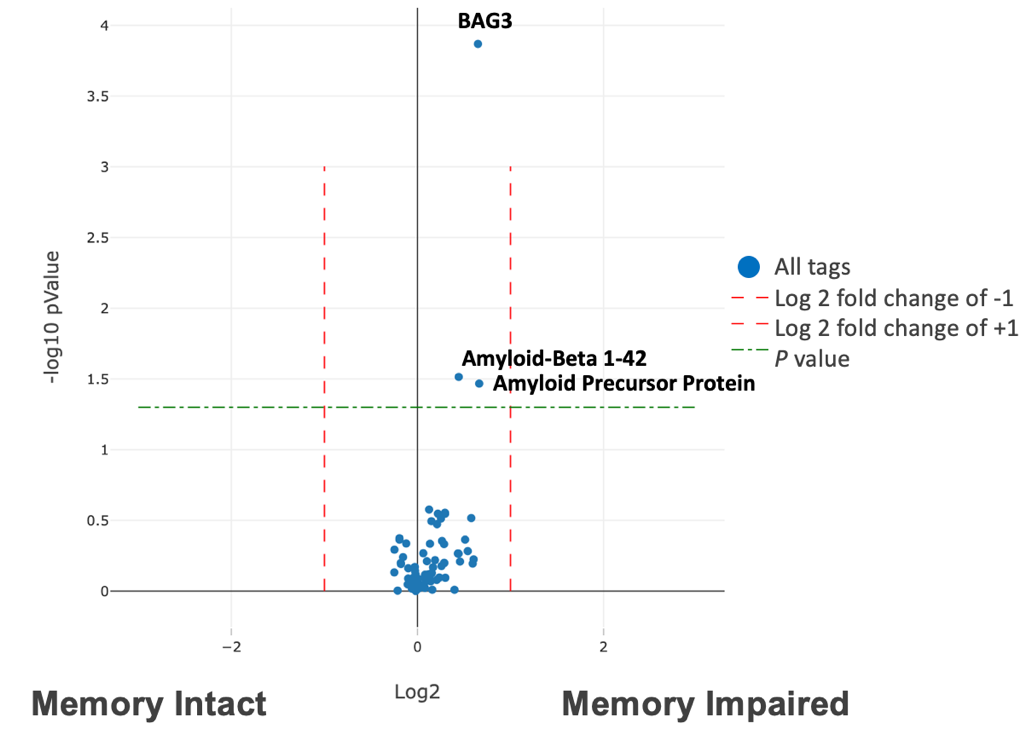
**

Volcano plots of differentially expressed proteins in hippocampal tissues from the first series (A) and second series (B) of TLE patients.

# **Supplementary Table 1: Differentially expressed proteins in neocortical tissues from TLE patients (series 1)**

| **Target group membership/s** | **Target name** | **Log2** | ***P* value** |
| --- | --- | --- | --- |
| All Targets, Astrocyte | Vimentin | 0.37093403 | 0.0024456 |
| All Targets | Alpha-synuclein | 1.02725028 | 0.00346512 |
| All Targets, Cytoskeleton, AD | Tau | 0.75374567 | 0.00615957 |
| All Targets, Autophagosome Formation | ATG12 | 0.30835336 | 0.0066169 |
| All Targets, PD | Park5 | 0.89693026 | 0.00739328 |
| All Targets, Microglia | TMEM119 | 0.47806099 | 0.00765859 |
| All Targets | Synaptophysin | 1.2075243 | 0.01258139 |
| All Targets, AD | Amyloid-Beta 1-42 | 0.60050395 | 0.01791861 |
| All Targets | Tyrosine Hydroxylase | 0.66963763 | 0.01991514 |
| All Targets | Myelin basic protein | 1.14467974 | 0.02455684 |
| All Targets, Endothelia | CD31 | 0.60401893 | 0.02459831 |
| All Targets, Neuron, Cytoskeleton | MAP2 | 0.73077544 | 0.02947261 |
| All Targets, Autophagy Promotion, Lysosomal Biogenesis | TFEB | 0.42904094 | 0.03243069 |
| All Targets | Neurofilament light | 0.76119553 | 0.03820137 |
| All Targets, Microglia | CD11b | 0.55136417 | 0.043193 |
| All Targets, Astrocyte Activation | C4B | 0.51332017 | 0.04548114 |

# **Supplementary Table 2: Differentially expressed proteins in neocortical tissues from TLE patients (series 2)**

| **Target group membership/s** | **Target name** | **Log2** | ***P* value** |
| --- | --- | --- | --- |
| All Targets, A2 Astrocyte, Astrocyte | EMP1 | -0.4732122 | 0.00187152 |
| All Targets, Neuron | NeuN | 0.79853006 | 0.00351116 |
| All Targets, Neuron, Cytoskeleton | MAP2 | 0.54518332 | 0.01940642 |
| All Targets, Microglia | CTSD | 0.39865979 | 0.03928118 |
| All Targets | Phospho-Alpha-synuclein (S129) | -0.4170744 | 0.0399526 |
| All Targets | Neurofilament light | 0.487221 | 0.04086746 |
